# Supplementary material for: Cytotoxicity and Bioactivity of Dental Pulp-Capping Agents towards Human Tooth-Pulp Cells: A Systematic Review of In-Vitro Studies and Meta-Analysis of Randomized and Controlled Clinical Trials
Source: Materials (Basel). 2020 Jun 12;13(12):2670. doi: 10.3390/ma13122670 (PMC7345102; doi:10.3390/ma13122670)
Supplement: Supplementary file 1 [file materials-13-02670-s001.pdf]

## *Pubmed* search strategy (after adaptation to *Embase* terms) by May 1, 2020

### 1- First concept: biocompatibility or pulp-tissue reaction. Results: 10,487,547

"Materials testing"[Mesh] OR materials-testing [tiab] OR biocompatibility-testing [tiab] OR biocompatib* [tiab] OR bioactive [tiab] OR bioactivity [tiab] OR “Cell survival” [mesh] OR survival [tiab] OR viability [tiab] OR cell-growth [tiab] OR “Cell proliferation” [Mesh] OR cell-proliferation [tiab] OR “Cell movement” [mesh] OR migration [tiab] OR movement [tiab] OR invasion [tiab] OR transwell [tiab] OR “Cell differentiation” [mesh] OR differentiation [tiab] OR cell-attachement [tiab] OR cell-adhesion [tiab] OR "Cytotoxicity, Immunologic"[Mesh] OR "Cytotoxicity Tests, Immunologic"[Mesh] OR "Toxicity Tests"[Mesh] OR toxicity-test* [tiab] OR cytotoxic* [tiab] OR "toxicity" [Subheading] OR toxicit* [tiab] OR toxicity-assay* [tiab] OR "Mutagenicity Tests"[Mesh] OR mutagenicity [tiab] OR genotoxic* [tiab] OR "Inflammation"[Mesh:NoExp] OR inflammation [tiab] OR "Inflammation Mediators"[Mesh] OR inflammation-mediator* [tiab] OR inflammation-marker* [tiab] OR "Cytokines"[Mesh:NoExp] OR cytokine* [tiab] OR "DNA Damage"[Mesh] OR dna-damage* [tiab] OR dna-break* [tiab] OR dna-fragmentation* [tiab] OR "Reactive Oxygen Species"[Mesh:NoExp] OR reactive-oxygen-specie* [tiab] OR "Complement Activation"[Mesh] OR complement-activation [tiab] OR Angiogenesis [tiab] OR "Gene Expression"[Mesh:NoExp] OR gene-expression* [tiab] OR expression* [tiab] OR “Cell Death"[Mesh:NoExp] OR cell-death [tiab] OR necrosis [tiab] OR apoptosis [tiab] OR pulp-tissue-response* [tiab] OR response* [tiab] OR reaction* [tiab] OR hard-tissue-formation [tiab] OR hard-tissue* [tiab] OR formation [tiab] OR barrier* [tiab] OR Dentin-bridge* [tiab] OR bridge* [tiab] OR osteodentin [tiab] OR Tertiary-dentin* [tiab] OR reparative-dentin* [tiab] OR reactionary-dentin* [tiab] OR Dentin-regenerat* [tiab] OR dentin-repair* [tiab] OR repair [tiab] OR regeneration [tiab] OR "Dentinogenesis"[Mesh] OR Dentinogenesis [tiab] OR maturogenesis [tiab] OR revitalization [tiab] OR apexogenesis [tiab] OR Angiogenesis [tiab] OR "Odontoblasts"[Mesh] OR Odontoblast* [tiab] OR "Dental Pulp Diseases"[Mesh] OR dental-pulp-disease* [tiab] OR pulp-necros* [tiab] OR "Pulpitis"[Mesh] OR pulpitis [tiab] OR "Periapical Abscess"[Mesh] OR abscess* [tiab] OR calcification* [tiab] OR "Dental Pulp Cavity"[Mesh] OR pulp-exposure* [tiab] OR pulp-cavit* [tiab] OR lesion* [tiab] OR Pulp-damage* [tiab] OR foci [tiab] OR mineralization [tiab] OR calcium-nodule* [tiab] OR pulp-stone* [tiab] OR "Pulpitis"[Mesh] OR pulpitis [tiab] OR inflammation [tiab] OR “Wound healing” [mesh:noExp] OR healing [tiab] OR success* [tiab]

### 2- Second concept: pulp-capping materials or treatments. Results: 559,789

((("Pulp Capping and Pulpectomy Agents"[Mesh] OR pulp-capping-material* [tiab] OR pulp-capping-agent* [tiab] OR biomedical-and-dental-material* [tiab] OR pulp-cap* [tiab] OR “Biomimetic materials” [Mesh:noExp] OR biomimetic-material* [tiab] OR “Biocompatible Materials” [Pharmacological Action] OR biocompatible-material* [tiab] OR Bioactive-material* [tiab] OR bioactive-cement* [tiab] OR "Biocompatible Materials"[Mesh:NoExp] OR biocompatible-material* [tiab] OR biomaterial* [tiab] OR bioactive-material* [tiab] OR “dental cements” [mesh] OR dental-cement* [tiab] OR dental-material* [tiab] OR tooth-cement* [tiab] OR silicates [Mesh] OR silicates [tiab] OR "Silicate Cement"[Mesh] OR silicate-cement* [tiab] OR silicate-based [tiab] OR tricalcium silicate [Supplementary Concept] OR "calcium silicate" [Supplementary Concept] OR calcium-silicate* [tiab] OR Ca3SiO5 [tiab] OR Biodentine [tiab] OR “Accelerated Portland cement” [Supplementary concept] OR Portland-cement* [tiab] OR “mineral trioxide aggregate” [Supplementary concept] OR mineral-trioxide-aggregate* [tiab] OR MTA [tiab] OR hydraulic-cement* [tiab] OR calcium-silicate-cement* [tiab] OR "Calcium Aluminosilicate"[Mesh] OR "aluminosilicate" [Supplementary Concept] OR calcium-aluminosilicate* [tiab] OR aluminosilicate* [tiab] OR "iRoot SP" [Supplementary Concept] OR "iRoot BP Plus" [Supplementary Concept] OR iRoot-BP-Plus [tiab] OR iRoot [tiab] OR "EndoSequence root repair material" [Supplementary Concept] OR EndoSequence-root-repair-material* [tiab] OR "BioAggregate" [Supplementary Concept] OR "DiaRoot BioAggregate" [Supplementary Concept] OR Bioaggregate [tiab] OR MTA [tiab] OR Theracal [tiab] OR Tech-Biosealer [tiab] OR “Endocem” [Supplementary concept] OR Endocem [tiab] OR Bio-C repair [tiab] OR Medcem [tiab] OR calcium-enriched-mixture [tiab] OR NEC [tiab] OR CEM [tiab] OR NeoMTA [tiab] OR Quick-set [tiab] OR TCS [tiab] OR NEX [tiab] OR Phosphopullulan [tiab] OR Biocal [tiab] OR IRM [tiab] OR “Calcium Compounds"[Mesh:NoExp] OR calcium-compound* [tiab] OR "Calcium Fluoride"[Mesh] OR calcium-fluoride* [tiab] OR "Calcium Hydroxide"[Mesh] OR calcium-hydroxide* [tiab] OR portlandite [tiab] OR pozzolan* [tiab] OR calcium-derivative* [tiab] OR "Calcium Phosphates"[Mesh] OR "whitlockite" [Supplementary Concept] OR calcium-phosphate [tiab] OR "beta-tricalcium phosphate" [Supplementary Concept] OR beta-tricalcium-phosphate* [tiab] OR "alpha-tricalcium phosphate" [Supplementary Concept] OR alpha-tricalcium-phosphate* [tiab] OR "tricalcium phosphate" [Supplementary Concept] OR tricalcium-phosphate* [tiab] OR “hydroxyapatites” [mesh] OR hydroxyapatite* [tiab] OR apatite* [tiab] OR “durapatite” [Mesh] OR durapatite [tiab] OR bioceramic* [tiab] OR “zinc oxide” [Mesh] OR zinc-oxide* [tiab] OR “zinc oxide-eugenol cement” [Mesh] OR zinc-oxide-eugenol-cement* [tiab] OR caryosan [tiab]))) OR ((((“composite resins” [Mesh] OR composite-resin* [tiab] OR resin-modified-material* [tiab] OR resin-based-material* [tiab] OR "Resin Cements"[Mesh] OR resin-cement* [tiab] OR "Epoxy Resins"[Mesh] OR epoxy-resin* [tiab] OR carisolv [tiab] OR "Bisphenol A-Glycidyl Methacrylate"[Mesh] OR Bisphenol-A-Glycidyl-Methacrylate* [tiab] OR methacrylate* [tiab] OR Bis-GMA [tiab] OR "Acrylic Resins"[Mesh] OR acrylic-resin* [tiab] OR "hydroxyethyl methacrylate" [Supplementary Concept]))) OR ((hydroxyethyl-methacrylate* [tiab] OR bisphenol-A-bis(2-hydroxypropyl)-ether-dimethacrylate* [tiab] OR methacrylic-acid-derivative* [tiab] OR 2-hydroxyethyl-methacrylate* [tiab] OR "Dentin-Bonding Agents"[Mesh] OR dentin-bonding-agent* [tiab] OR adhesive* [tiab] OR bonding [tiab] OR primer* [tiab] OR etch* [tiab] OR “Glass ionomer cements” [mesh] OR glass-ionomer* [tiab] OR cermet [tiab] OR Bioglass [Supplementary Concept] OR Bioglass [tiab] OR chemfil [tiab] OR ketac [tiab] OR bioactive-glass* [tiab])))

### 3- Third concept: (human) dental pulp cells or (human) dental pulp tissue or teeth. Results: 30,511

"Dental Pulp"[Mesh] OR dental-pulp* [tiab] OR tooth-pulp* [tiab] OR pulp-tissue* [tiab] pulp* [tiab] OR Pulp-fibroblast* [tiab] OR dental-pulp-stem-cell* [tiab] OR apical-papilla [tiab] OR stem-cells-from-human-exfoliated-deciduous-teeth [tiab] OR "Odontoblasts"[Mesh] OR odontoblast* [tiab] OR "Dental Pulp Cavity"[Mesh] OR dental-pulp-cavit* [tiab] OR pulp-chamber [tiab] OR dentin-pulp-complex [tiab] OR pulp-exposure* [tiab] OR "Dental Pulp"[Mesh] OR dental-pulp* [tiab] OR “Pulpotomy” [Mesh] OR pulpotom* [tiab] OR Vital-pulp-therap* [tiab] OR pulp-therap* [tiab] OR “Dental pulp capping” [Mesh] OR pulp-cap* [tiab] OR “Regenerative endodontics” [mesh] OR regenerative-endodontic* [tiab] OR endodontic-procedure* [tiab]

**Combination: #1 AND #2 AND #3: 6,490 results**

Figure S1: Details of the *PubMed* electronic search strategy.

## *Embase* search strategy by May 1, 2020

### 1- First concept: biocompatibility or pulp-tissue reaction. Results: 13,050,184

'materials testing'/exp OR 'materials testing':ti,ab,kw OR 'biocompatibility'/exp OR 'biocompatibility testing':ti,ab,kw OR 'biocompatib*':ti,ab,kw OR 'biocompatibility':ti,ab,kw OR 'bioactive':ti,ab,kw OR 'bioactivity':ti,ab,kw OR 'cell viability'/de OR 'cell survival'/de OR 'survival':ti,ab,kw OR 'viability':ti,ab,kw OR 'cell growth':ti,ab,kw OR 'cell proliferation'/exp OR 'cell proliferation':ti,ab,kw OR 'cell motion'/exp OR 'migration':ti,ab,kw OR 'movement':ti,ab,kw OR 'invasion':ti,ab,kw OR 'transwell':ti,ab,kw OR 'cell differentiation'/de OR 'differentiation':ti,ab,kw OR 'cell attachement':ti,ab,kw OR 'cell adhesion'/de OR 'cell adhesion':ti,ab,kw OR 'cytotoxicity'/de OR 'cytotoxicity test'/exp OR 'cytotoxicity assay'/de OR 'cytotox*':ti,ab,kw OR 'toxicity'/de OR 'toxicity test*':ti,ab,kw OR 'toxicity assay*':ti,ab,kw OR 'toxicit*':ti,ab,kw OR 'mutagen testing'/de OR 'mutagenicity'/exp OR 'mutagenicity':ti,ab,kw OR 'mutagen testing':ti,ab,kw OR 'genotoxic*':ti,ab,kw OR 'inflammation'/de OR 'inflammation':ti,ab,kw OR 'Inflammation mediator*':ti,ab,kw OR 'Inflammation marker*':ti,ab,kw OR 'cytokine'/de OR 'cytokine*':ti,ab,kw OR 'DNA damage'/exp OR 'dna damage*':ti,ab,kw OR 'dna break*':ti,ab,kw OR 'dna fragmentation*':ti,ab,kw OR 'reactive oxygen metabolite'/exp OR 'reactive oxygen metabolite*':ti,ab,kw OR 'Reactive Oxygen Specie*':ti,ab,kw OR 'complement activation'/exp OR 'complement activation':ti,ab,kw OR 'angiogenesis'/de OR 'Angiogenesis':ti,ab,kw OR 'gene expression'/de OR 'gene expression*':ti,ab,kw OR 'expression*':ti,ab,kw OR 'cell death'/exp OR 'necrosis'/de OR 'apoptosis'/de OR 'cell death':ti,ab,kw OR 'necrosis':ti,ab,kw OR 'apoptosis':ti,ab,kw OR 'pulp tissue response*':ti,ab,kw OR 'response*':ti,ab,kw OR 'reaction*':ti,ab,kw OR 'hard tissue formation':ti,ab,kw OR 'hard tissue*':ti,ab,kw OR 'formation':ti,ab,kw OR 'barrier*':ti,ab,kw OR 'Dentin bridge*':ti,ab,kw OR 'bridge*':ti,ab,kw OR 'osteodentin':ti,ab,kw OR 'tertiary dentin*':ti,ab,kw OR 'reparative dentin*':ti,ab,kw OR 'reactionary dentin*':ti,ab,kw OR 'Dentin regenerat*':ti,ab,kw OR 'dentin repair*':ti,ab,kw OR 'repair':ti,ab,kw OR 'regeneration':ti,ab,kw OR 'dentinogenesis'/exp OR 'Dentinogenesis':ti,ab,kw OR 'maturogenesis':ti,ab,kw OR 'revitalization':ti,ab,kw OR 'apexogenesis':ti,ab,kw OR 'angiogenesis'/de or 'angiogenesis':ti,ab,kw OR 'odontoblast'/exp OR 'odontoblast*':ti,ab,kw OR 'tooth pulp disease'/exp OR 'tooth pulp disease*':ti,ab,kw OR 'dental pulp disease*':ti,ab,kw OR 'pulp necros*':ti,ab,kw OR 'pulpitis'/exp OR 'pulpitis':ti,ab,kw OR 'tooth abscess'/exp OR 'abscess*':ti,ab,kw OR 'calcification*':ti,ab,kw OR 'dental pulp cavity'/exp OR 'pulp exposure*':ti,ab,kw OR 'pulp cavit*':ti,ab,kw OR 'lesion*':ti,ab,kw OR 'Pulp-damage*':ti,ab,kw OR 'Foci':ti,ab,kw OR 'mineralization':ti,ab,kw OR 'calcium nodule*':ti,ab,kw OR 'pulp stone*':ti,ab,kw OR 'pulpitis'/exp OR 'pulpitis':ti,ab,kw OR 'inflammation':ti,ab,kw OR 'wound healing'/de OR 'wound healing':ti,ab,kw OR 'healing':ti,ab,kw OR 'success*':ti,ab,kw

### 2- Second concept: pulp-capping materials or treatments. Results: 385,673

'biomedical and dental materials'/de OR 'biomedical and dental material*':ti,ab,kw OR 'pulp Capping and Pulpectomy Agent*':ti,ab,kw OR 'pulp capping material*':ti,ab,kw OR 'pulp capping agent*':ti,ab,kw OR 'pulp cap*':ti,ab,kw OR 'biomimetic material'/exp OR 'biomimetic material*':ti,ab,kw OR 'biomaterial'/de OR 'biomaterial*':ti,ab,kw OR 'biocompatible material*':ti,ab,kw OR 'Bioactive material*':ti,ab,kw OR 'bioactive cement*':ti,ab,kw OR 'tooth cement'/exp OR 'tooth cement*':ti,ab,kw OR 'dental cement*':ti,ab,kw OR 'dental material*':ti,ab,kw OR 'silicate'/exp OR 'silicate':ti,ab,kw OR 'silicate cement'/exp OR 'silicate cement*':ti,ab,kw OR 'silicate based':ti,ab,kw OR 'tricalcium silicate':ti,ab,kw OR 'calcium silicate'/exp OR 'calcium silicate*':ti,ab,kw OR 'Ca3SiO5':ti,ab,kw OR 'Biodentine':ti,ab,kw OR 'Accelerated Portland cement':ti,ab,kw OR 'Portland cement*':ti,ab,kw OR 'mineral trioxide aggregate':ti,ab,kw OR 'MTA':ti,ab,kw OR 'hydraulic cement*':ti,ab,kw OR 'calcium silicate cement*':ti,ab,kw OR 'aluminosilicate calcium'/exp OR 'aluminosilicate calcium':ti,ab,kw OR 'calcium aluminosilicate*':ti,ab,kw OR 'aluminosilicate*':ti,ab,kw OR 'iRoot BP Plus':ti,ab,kw OR 'iRoot':ti,ab,kw OR 'EndoSequence root repair material*':ti,ab,kw OR 'BioAggregate' OR 'MTA':ti,ab,kw OR 'Theracal':ti,ab,kw OR 'Tech Biosealer':ti,ab,kw OR 'Endocem' OR 'Endocem':ti,ab,kw OR 'Bio C repair':ti,ab,kw OR 'Medcem':ti,ab,kw OR 'calcium enriched mixture':ti,ab,kw OR 'NEC':ti,ab,kw OR 'CEM':ti,ab,kw OR 'NeoMTA':ti,ab,kw OR 'Quick set':ti,ab,kw OR 'TCS':ti,ab,kw OR 'NEX':ti,ab,kw OR 'Phosphopullulan':ti,ab,kw OR 'Biocal':ti,ab,kw OR 'IRM':ti,ab,kw OR 'calcium derivative'/exp OR 'calcium derivative*':ti,ab,kw OR 'calcium compound*':ti,ab,kw OR 'calcium fluoride'/exp OR 'calcium fluoride':ti,ab,kw OR 'calcium hydroxide'/exp OR 'calcium-hydroxide*':ti,ab,kw OR 'portlandite':ti,ab,kw OR 'pozzolan*':ti,ab,kw 'calcium phosphate'/exp OR 'calcium phosphate*':ti,ab,kw OR 'tricalcium phosphate*':ti,ab,kw OR 'beta tricalcium phosphate*':ti,ab,kw OR 'alpha tricalcium phosphate*':ti,ab,kw OR 'hydroxyapatite'/exp OR 'hydroxyapatite*':ti,ab,kw OR 'apatite*':ti,ab,kw OR 'durapatite':ti,ab,kw OR 'bioceramics'/exp OR 'bioceramic*':ti,ab,kw OR 'zinc oxide eugenol'/exp OR 'zinc oxide*':ti,ab,kw OR 'zinc oxide cement*':ti,ab,kw OR 'caryosan':ti,ab,kw OR 'composite resin*':ti,ab,kw OR 'resin modified material*':ti,ab,kw OR 'resin based material*':ti,ab,kw OR 'resin cement'/exp OR 'resin cement*':ti,ab,kw OR 'epoxy resin'/exp OR 'epoxy resin*':ti,ab,kw OR 'calcium phosphate'/exp OR 'calcium phosphate*':ti,ab,kw OR 'tricalcium phosphate*':ti,ab,kw OR 'beta tricalcium phosphate*':ti,ab,kw OR 'alpha tricalcium phosphate*':ti,ab,kw OR 'hydroxyapatite'/exp OR 'hydroxyapatite*':ti,ab,kw OR 'apatite*':ti,ab,kw OR 'durapatite':ti,ab,kw OR 'bioceramics'/exp OR 'bioceramic*':ti,ab,kw OR 'zinc oxide eugenol'/exp OR 'zinc oxide*':ti,ab,kw OR 'zinc oxide cement*':ti,ab,kw OR 'caryosan':ti,ab,kw OR 'composite resin*':ti,ab,kw OR 'resin modified material*':ti,ab,kw OR 'resin based material*':ti,ab,kw OR 'resin cement'/exp OR 'resin cement*':ti,ab,kw OR 'epoxy resin'/exp OR 'epoxy resin*':ti,ab,kw OR 'carisolv':ti,ab,kw OR 'bisphenol A bis(2 hydroxypropyl) ether dimethacrylate'/exp OR 'bisphenol A bis(2 hydroxypropyl) ether dimethacrylate*':ti,ab,kw OR 'Bisphenol A Glycidyl Methacrylate*':ti,ab,kw OR 'methacrylic acid derivative'/exp OR 'methacrylic acid derivative*':ti,ab,kw OR 'methacrylate*':ti,ab,kw OR 'Bis GMA':ti,ab,kw OR 'acrylic acid resin'/exp OR 'acrylic resin*':ti,ab,kw OR '2 hydroxyethyl methacrylate'/exp OR 'hydroxyethyl methacrylate*':ti,ab,kw OR 'dentin bonding agent'/exp OR 'dentin bonding agent*':ti,ab,kw OR 'adhesive*':ti,ab,kw OR 'bonding':ti,ab,kw OR 'primer*':ti,ab,kw OR 'etch*':ti,ab,kw OR 'glass ionomer'/exp OR 'glass ionomer*':ti,ab,kw OR 'cermet':ti,ab,kw OR 'Bioglass*':ti,ab,kw OR 'Bioactive glass*':ti,ab,kw OR 'chemfil':ti,ab,kw OR 'ketac':ti,ab,kw

### 3- Third concept: (human) dental pulp cells or (human) dental pulp tissue or teeth. Results: 61,719

'tooth pulp'/exp OR 'tooth pulp*':ti,ab,kw OR 'dental pulp*':ti,ab,kw OR 'pulp tissue*':ti,ab,kw OR 'pulp*':ti,ab,kw OR 'Pulp fibroblast*':ti,ab,kw OR 'dental pulp stem cell'/exp OR 'dental pulp stem cell*':ti,ab,kw OR 'apical papilla':ti,ab,kw OR 'stem cells from human exfoliated deciduous teeth':ti,ab,kw OR 'stem cells from apical papilla':ti,ab,kw OR 'odontoblast'/exp OR 'odontoblast*':ti,ab,kw OR 'dental pulp cavity'/exp OR 'dental pulp cavit*':ti,ab,kw OR 'pulp chamber':ti,ab,kw OR 'dentin pulp complex*':ti,ab,kw OR 'pulp exposure*':ti,ab,kw OR 'pulpotomy'/exp OR 'pulpotom*':ti,ab,kw OR 'Vital pulp therap*':ti,ab,kw OR 'pulp therap*':ti,ab,kw OR 'dental pulp capping'/exp OR 'pulp cap*':ti,ab,kw OR 'endodontic procedure'/exp OR 'endodontic procedure*':ti,ab,kw OR 'regenerative endodontics'/exp OR 'regenerative-endodontic*':ti,ab,kw

**Combination: #1 AND #2 AND #3: 4,369 results**

Figure S2: Details of the *EMBASE* electronic search strategy.

## *Web of Science* search strategy by May 1, 2020

### 1- First concept: biocompatibility or pulp-tissue reaction. Results: 16,932,222

"Materials testing" OR "biocompatibility testing" OR "biocompatib*" OR "bioactive" OR "bioactivity" OR "Cell survival" OR "survival" OR "viability" OR "cell growth" OR "Cell proliferation" OR "Cell movement" OR "migration" OR "movement" OR "invasion" OR "transwell" OR "Cell differentiation" OR "differentiation" OR "cell attachement" OR "cell adhesion" OR "toxicity test*" OR "cytotoxic*" OR "toxicit*" OR "toxicity assay*" OR "Mutagenicity Tests" OR "mutagenicity" OR "genotoxic*" OR "Inflammation" OR "Inflammation Mediator*" OR "inflammation marker*" OR "Cytokine*" OR "DNA Damage" OR "dna break*" OR "dna fragmentation*" OR "Reactive Oxygen Species" OR "Complement Activation" OR "Angiogenesis" OR "gene expression*" OR "expression" OR "Cell Death" OR "necrosis" OR "apoptosis" OR "pulp tissue response*" OR "response*" OR "reaction*" OR "hard tissue formation" OR "hard tissue*" OR "formation" OR "barrier*" OR "Dentin bridge*" OR "bridge*" OR "osteodentin" OR "Tertiary dentin*" OR "reparative dentin*" OR "reactionary dentin*" OR "Dentin regenerat*" OR "dentin repair*" OR "repair" OR "regeneration" OR "Dentinogenesis" OR "maturogenesis" OR "revitalization" OR "apexogenesis" OR "Angiogenesis" OR "Odontoblast*" OR "Dental Pulp Disease*" OR "pulp necros*" OR "Pulpitis" OR "Periapical Abscess*" OR "abscess*" OR "calcification*" OR "Dental Pulp Cavit*" OR "pulp exposure*" OR "pulp cavit*" OR "lesion*" OR "Pulp damage*" OR "foci" OR "mineralization" OR "calcium nodule*" OR "pulp stone*" OR "inflammation" OR "Wound healing" OR "healing" OR "success*"

### 2- Second concept: pulp-capping materials or treatments. Results: 1,067,072

((("Pulp Capping and Pulpectomy Agents" OR "pulp capping material*" OR "pulp capping agent*" OR "biomedical and dental material*" OR "pulp cap*" OR "Biomimetic materials" OR "biomimetic material*" OR "Biocompatible Materials" OR "biocompatible material*" OR "Bioactive material*" OR " bioactive cement*" OR "Biocompatible Materials" OR "biocompatible material*" OR "biomaterial*" OR " bioactive material*" OR "dental cements" OR "dental cement*" OR "dental material*" OR " tooth cement*" OR "silicates" OR "silicates" OR "silicate cement*" OR "silicate based" OR "tricalcium silicate" OR "calcium silicate" OR "calcium silicate*" OR "Ca3SiO5" OR "Biodentine" OR "Accelerated Portland cement" OR "Portland cement*" OR "mineral trioxide aggregate" OR "mineral trioxide aggregate*" OR "MTA" OR "hydraulic cement*" OR "calcium silicate cement*" OR "Calcium Aluminosilicate" OR "aluminosilicate" OR "calcium aluminosilicate*" OR "aluminosilicate*" OR "iRoot SP" OR "iRoot BP Plus" OR " iRoot BP Plus" OR " iRoot" OR "EndoSequence root repair material" OR "EndoSequence root repair material*" OR "BioAggregate" OR "DiaRoot BioAggregate" OR "Bioaggregate" OR "MTA" OR "Theracal" OR "Tech Biosealer" OR "Endocem" OR "Bio C repair" OR "Medcem" OR "calcium enriched mixture" OR "NEC" OR "CEM" OR "NeoMTA" OR "Quick set" OR "TCS" OR "NEX" OR "Phosphopullulan" OR "Biocal" OR "IRM" OR "Calcium Compounds" OR "calcium compound*" OR "Calcium Fluoride" OR "Calcium Hydroxide" OR "Potlandite" OR "pozzolan*" OR "calcium derivative*" OR "Calcium Phosphates" OR "whitlockite" OR "calcium phosphate" OR "beta tricalcium phosphate" OR "beta tricalcium phosphate*" OR "alpha tricalcium phosphate" OR "alpha tricalcium phosphate*" OR "tricalcium phosphate" OR "tricalcium phosphate*" OR "hydroxyapatites" OR " hydroxyapatite*" OR "apatite*" OR "durapatite" OR "bioceramic*" OR " zinc oxide*" OR "zinc oxide eugenol cement*" OR "caryosan" ))) OR (((("composite resins" OR "composite resin*" OR "resin modified material*" OR "resin based material*" OR "Resin Cements" OR "resin cement*" OR "epoxy resin*" OR "carisolv" OR "Bisphenol A Glycidyl Methacrylate*" OR "methacrylate*" OR "Bis GMA" OR "acrylic resin*" OR "hydroxyethyl methacrylate"))) OR (("hydroxyethyl methacrylate*" OR "bisphenol A bis(2 hydroxypropyl) ether dimethacrylate*" OR "methacrylic acid derivative*" OR "2 hydroxyethyl methacrylate*" OR "Dentin Bonding Agents" OR "dentin bonding agent*" OR "adhesive*" OR "bonding" OR "primer*" OR "etch*" OR "Glass ionomer cements" OR "glass ionomer*" OR "cermet" OR "Bioglass" OR "Bioglass" OR "chemfil" OR "ketac" OR "bioactive glass*" )))

### 3- Third concept: (human) dental pulp cells or (human) dental pulp tissue or teeth. Results: 97,319

"Dental Pulp" OR " dental pulp*" OR " tooth pulp*" OR " pulp tissue*" OR "pulp*" OR "Pulp fibroblast*" OR " dental pulp stem cell*" OR "apical papilla" OR " stem cells from human exfoliated deciduous teeth" OR "odontoblast*" OR "Dental Pulp Cavity" OR "dental pulp cavit*" OR "pulp chamber" OR "dentin pulp complex" OR "pulp exposure*" OR "Dental Pulp" OR "dental pulp*" OR "pulpotom*" OR "Vital pulp therap*" OR "pulp therap*" OR "Dental pulp capping" OR "pulp cap*" OR "Regenerative endodontics" OR "regenerative endodontic*" OR "endodontic procedure*"

**Combination: #1 AND #2 AND #3: 5,682 results**

Figure S3: Details of the *WEB OF SCIENCE* electronic search strategy.

Table S1. Excluded full-text in vivo studies and the reasons for exclusion.

| Number | Studies | Reason for exclusion |
|---|---|---|
| 1. | Subay RK, Asci S. Human pulpal response to hydroxyapatite and a calcium hydroxide material as direct capping agents. Oral Surg Oral Med Oral Pathol. 1993;76(4):485-92. | No Ethical Commission approval mentioned<br>Not clear if bridges complete or not |
| **2.** | Subay RK, Suzuki S, Suzuki S, Kaya H, Cox CF. Human pulp response after partial pulpotomy with 2 calcium hydroxide products. Oral Surgery Oral Medicine Oral Pathology Oral Radiology and Endodontics. 1995;80(3):330-7. | No Ethical Commission approval mentioned<br>Not clear if bridges complete or not |
| **3.** | Yoshiba K, Yoshiba N, Nakamura H, Iwaku M, Ozawa H. Immunolocalization of fibronectin during reparative dentinogenesis in human teeth after pulp capping with calcium hydroxide. J Dent Res. 1996;75(8):1590-7. | No H&E staining<br>No analysis of bridge formation |

| | | |
|---|---|---|
| **4.** | Pereira JC, Segala AD, Costa CAS. Human pulpal response to direct pulp capping with an adhesive system. American Journal of Dentistry. 2000;13(3):139-47. | Not clear if bridges complete or not |
| **5.** | Aeinehchi M, Eslami B, Ghanbariha M, Saffar AS. Mineral trioxide aggregate (MTA) and calcium hydroxide as pulp-capping agents in human teeth: a preliminary report. Int Endod J. 2003;36(3):225-31. | Not clear if bridges complete or not<br>Some groups only 1 sample |
| **6.** | Hörsted-Bindslev P, Vilkinis V, Sidlauskas A. Direct capping of human pulps with a dentin bonding system or with calcium hydroxide cement. Oral Surgery, Oral Medicine, Oral Pathology, Oral Radiology, and Endodontics. 2003;96(5):591-600. | No rubber dam mentioned |
| **7.** | Murray PE, Smith AJ, Windsor LJ, Mjör IA. Remaining dentine thickness and human pulp responses. International endodontic journal. 2003;36(1):33-43. | No rubber dam mentioned<br>Retrospective study |
| **8.** | Scarano A, Manzon L, Di Giorgio R, Orsini G, Tripodi D, Piattelli A. Direct capping with four different materials in humans: histological analysis of odontoblast activity. J Endod. 2003;29(11):729-34. | No H&E staining<br>Not clear if bridges complete or not |
| **9.** | Accorinte Mde L, Loguercio AD, Reis A, Muench A, de Araujo VC. Response of human pulp capped with a bonding agent after bleeding control with hemostatic agents. Oper Dent. 2005;30(2):147-55. | Data of interest is the same as in other article from the same authors (*Dental Materials*) |
| **10.** | Sübay RK, Demirci M. Pulp tissue reactions to a dentin bonding agent as a direct capping agent. Journal of Endodontics. 2005;31(3):201-4. | Not clear if bridges complete or not |
| **11.** | Chacko V, Kurikose S. Human pulpal response to mineral trioxide aggregate (MTA): a histologic study. J Clin Pediatr Dent. 2006;30(3):203-9. | No rubber dam mentioned<br>Not clear if bridges complete or not |
| **12.** | Piva E, Tarquínio SBC, Demarco FF, Silva AF, de Araújo VC. Immunohistochemical expression of fibronectin and tenascin after direct pulp capping with calcium hydroxide. Oral Surgery, Oral Medicine, Oral Pathology, Oral Radiology, and Endodontics. 2006;102(4):e66-e71. | No H&E staining<br>Not clear if bridges complete or not |
| **13.** | Silva AF, Tarquinio SBC, Demarco FF, Piva E, Rivero ERC. The influence of haemostatic agents on healing of healthy human dental pulp tissue capped with calcium hydroxide. International Endodontic Journal. 2006;39(4):309-16. | No quantitative data is provided (descriptve study)<br>Not clear if bridges complete or not |
| **14.** | D'Arcangelo C, Di Nardo-Di Maio F, Patrono C, Caputi S. NOS evaluations in human dental pulp-capping with MTA and calcium-hydroxide. Int J Immunopathol Pharmacol. 2007;20(1 Suppl 1):27-32. | No H&E staining<br>No analysis of bridge formation |
| **15.** | Fernandes AM, Silva GAB, Lopes N, Napimoga MH, Benatti BB, | No H&E staining |

| | | |
|---|---|---|
| | Alves JB. Direct capping of human pulps with a dentin bonding system and calcium hydroxide: an immunohistochemical analysis. Oral Surgery Oral Medicine Oral Pathology Oral Radiology and Endodontology. 2008;105(3):385-90. | No analysis of bridge formation |
| **16.** | Barbosa AV, Sampaio GC, Gomes FA, de Oliveira DP, de Albuquerque DS, Sobral AP. Short-term analysis of human dental pulps after direct capping with portland cement. Open Dent J. 2009;3:31-5. | Case Series (not randomized or controlled clinical trial) |
| **17.** | Pereira SA, de Menezes FC, Rocha-Rodrigues DB, Alves JB. Pulp reactions in human teeth capped with self-etching or total-etching adhesive systems. Quintessence Int. 2009;40(6):491-6. | No direct pulp exposure<br>No rubber dam mentioned |
| **18.** | Zarrabi MH, Javidi M, Jafarian AH, Joushan B. Histologic assessment of human pulp response to capping with mineral trioxide aggregate and a novel endodontic cement. J Endod. 2010;36(11):1778-81. | Not clear if bridges complete or not |
| **19.** | Eskandarizadeh A, Shahpasandzadeh MH, Shahpasandzadeh M, Torabi M, Parirokh M. A comparative study on dental pulp response to calcium hydroxide, white and grey mineral trioxide aggregate as pulp capping agents. J Conserv Dent. 2011;14(4):351-5. | No rubber dam mentioned |
| **20.** | Fransson H, Petersson K, Davies JR. Dentine sialoprotein and Collagen I expression after experimental pulp capping in humans using Emdogain (R) Gel. International Endodontic Journal. 2011;44(3):259-67. | Data of interest is the same as in other article from the same authors (*Int End Journal 2005*) |
| **21.** | Shahravan A, Jalali SP, Torabi M, Haghdoost AA, Gorjestani H. A histological study of pulp reaction to various water/powder ratios of white mineral trioxide aggregate as pulp-capping material in human teeth: a double-blinded, randomized controlled trial. Int Endod J. 2011;44(11):1029-33. | Isolation with cotton rolls (no rubber dam mentioned) |
| **22.** | Zarrabi MH, Javidi M, Jafarian AH, Joushan B. Immunohistochemical expression of fibronectin and tenascin in human tooth pulp capped with mineral trioxide aggregate and a novel endodontic cement. J Endod. 2011;37(12):1613-8. | Data of interest is the same as in other article from the same authors (*J Endod 2010*)<br>Not clear if bridges complete or not |
| **23.** | Yoshiba N, Yoshiba K, Ohkura N, Hosoya A, Shigetani Y, Yamanaka Y, et al. Expressional alterations of fibrillin-1 during wound healing of human dental pulp. J Endod. 2012;38(2):177-84. | Not clear if bridges complete or not<br>No quantitative data is provided (descriptve study) |
| **24.** | Chandwani ND, Pawar MG, Tupkari JV, Yuwanati M. Histological evaluation to study the effects of dental amalgam and composite restoration on human dental pulp: An in vivo study. Medical Principles and Practice. 2013;23(1):40-4. | No direct pulp exposure |
| **25.** | Nosrat A, Peimani A, Asgary S. A preliminary report on histological outcome of pulpotomy with endodontic | Not clear if bridges complete or not<br>Bridge formation analized per root (not |

| | | |
|---|---|---|
| | biomaterials vs calcium hydroxide. Restor Dent Endod. 2013;38(4):227-33. | per tooth) |
| **26.** | Silva GA, Gava E, Lanza LD, Estrela C, Alves JB. Subclinical failures of direct pulp capping of human teeth by using a dentin bonding system. J Endod. 2013;39(2):182-9. | Not clear if bridges complete or not<br>No quantitative data is provided (descriptve study) |
| **27.** | Cebe F, Cobanoglu N, Ozdemir O. Response of exposed human pulp to application of a hemostatic agent and a self-etch adhesive. Journal of Adhesion Science and Technology. 2015;29(24):2719-30. | No rubber dam mentioned<br>50% samples with bacteria contamination<br>*unable to contact authors |
| **28.** | Eftimoska M, Apostolska S, Rendzhova V, Gjorgievska E, Stevanovic M, Ivanovski K, et al. Clinical and Histological Analyzes of the Response of the Pulp after Its Direct Capping with Calxyl, MTA and Biodentine. Research Journal of Pharmaceutical Biological and Chemical Sciences. 2015;6(4):1097-111. | No rubber dam mentioned<br>Not only "healthy" teeth used |
| **29.** | Haghgoo R, Asgary S, Mashhadi Abbas F, Montazeri Hedeshi R. Nano-hydroxyapatite and calcium-enriched mixture for pulp capping of sound primary teeth: a randomized clinical trial. Iran Endod J. 2015;10(2):107-11. | No rubber dam mentioned |
| **30.** | Nowicka A, Wilk G, Lipski M, Kolecki J, Buczkowska-Radlinska J. Tomographic Evaluation of Reparative Dentin Formation after Direct Pulp Capping with Ca(OH)2, MTA, Biodentine, and Dentin Bonding System in Human Teeth. J Endod. 2015;41(8):1234-40. | CBCT study<br>Not clear if bridges complete or not |
| **31.** | AlShwaimi E, Majeed A, Ali AA. Pulpal Responses to Direct Capping with Betamethasone/Gentamicin Cream and Mineral Trioxide Aggregate: Histologic and Micro-Computed Tomography Assessments. J Endod. 2016;42(1):30-5. | Not clear which type of MTA used<br>*unable to contact authors |
| **32.** | Bhagat D, Sunder RK, Devendrappa SN, Vanka A, Choudaha N. A comparative evaluation of ProRoot mineral trioxide aggregate and Portland cement as a pulpotomy medicament. J Indian Soc Pedod Prev Dent. 2016;34(2):172-6. | Not clear if bridges complete or not<br>60% failure rate with MTA<br>*unable to contact authors |
| **33.** | Bollu IP, Velagula LD, Bolla N, Kumar KK, Hari A, Thumu J. Histological evaluation of mineral trioxide aggregate and enamel matrix derivative combination in direct pulp capping: An in vivo study. J Conserv Dent. 2016;19(6):536-40. | Not clear if bridges complete or not |
| **34.** | Shameem A, Muliyar S, Thankachan RP, Kalliath JT, Mangalath U, Mangalath S. Study to evaluate the Efficacy of Resin-modified Glass lonomer Cement Liner as a Direct Pulp Capping Material. J Contemp Dent Pract. 2018;19(9):1065-71. | Not clear if bridges complete or not |
| **35.** | Arafa A, Kenawi LMM, Issa N. Assessment of reparative hard tissue formation after direct pulp capping with Biodentine versus mineral trioxide aggregate. Endo-Endodontic Practice | CBCT study |

| | | |
|---|---|---|
| | Today. 2019;13(3):227-36. | |
| **36.** | Chen CA, Chen YL, Huang JS, Huang GT, Chuang SF. Effects of Restorative Materials on Dental Pulp Stem Cell Properties. J Endod. 2019;45(4):420-6. | No direct pulp exposure |
